# Delineation of molecular findings by whole-exome sequencing for suspected cases of paediatric-onset mitochondrial diseases in the Southern Chinese population

**DOI:** 10.1186/s40246-020-00278-0

**Published:** 2020-09-10

**Authors:** Mandy H.Y. Tsang, Anna K.Y. Kwong, Kate L.S. Chan, Jasmine L.F. Fung, Mullin H.C. Yu, Christopher C.Y. Mak, Kit-San Yeung, Richard J.T. Rodenburg, Jan A.M. Smeitink, Rachel Chan, Thomas Tsoi, Joannie Hui, Shelia S.N Wong, Shuk-Mui Tai, Victor C.M. Chan, Che-Kwan Ma, Sharon T.H. Fung, Shun-Ping Wu, W.K. Chak, Brian H.Y. Chung, Cheuk-Wing Fung

**Affiliations:** 1 Department of Paediatrics & Adolescent Medicine, LKS Faculty of Medicine, The University of Hong Kong, Hong Kong, SAR China; 2 Radboud Center for Mitochondrial Medicine, Department of Paediatrics, Radboud Institute for Molecular Life Sciences, Radboud University Nijmegen Medical Centre, Nijmegen, The Netherlands; 3 Department of Paediatrics, Centro Hospitalar Conde de São Januário (CHCSJ) Hospital, SAR, Macau, China; 4 Department of Paediatrics and Adolescent Medicine, Hong Kong Children’s Hospital, Doctors’ Office, 9/F, Tower B, 1 Shing Cheong Road, Kowloon Bay, Kowloon, Hong Kong, SAR China; 5 Department of Paediatrics & Adolescent Medicine, Pamela Youde Nethersole Eastern Hospital, Hong Kong, SAR China; 6 Department of Paediatrics and Adolescent Medicine, United Christian Hospital, Hong Kong, SAR China; 7 Department of Paediatrics, Kwong Wah Hospital, Hong Kong, SAR China; 8 Department of Paediatrics and Adolescent Medicine, Queen Elizabeth Hospital, Hong Kong, SAR China; 9 Department of Paediatrics and Adolescent Medicine, Tuen Mun Hospital, Hong Kong, SAR China; 10 Department of Pediatrics and Adolescent Medicine, Queen Mary Hospital, Room 115, 1/F, New Clinical Building, 102 Pokfulam Road, Hong Kong, SAR China; 11 Department of Paediatrics & Adolescent Medicine, Duchess of Kent Children’s Hospital, Hong Kong, SAR China

**Keywords:** Mitochondrial disease, Paediatrics, Whole-exome sequencing

## Abstract

**Background:**

Mitochondrial diseases (MDs) are a group of clinically and genetically heterogeneous disorders characterized by defects in oxidative phosphorylation. Since clinical phenotypes of MDs may be non-specific, genetic diagnosis is crucial for guiding disease management. In the current study, whole-exome sequencing (WES) was performed for our paediatric-onset MD cohort of a Southern Chinese origin, with the aim of identifying key disease-causing variants in the Chinese patients with MDs.

**Methods:**

We recruited Chinese patients who had paediatric-onset MDs and a minimum mitochondrial disease criteria (MDC) score of 3. Patients with positive target gene or mitochondrial DNA sequencing results were excluded. WES was performed, variants with population frequency ≤ 1% were analysed for pathogenicity on the basis of the American College of Medical Genetics and Genomics guidelines.

**Results:**

Sixty-six patients with pre-biopsy MDC scores of 3–8 were recruited. The overall diagnostic yield was 35% (23/66). Eleven patients (17%) were found to have mutations in MD-related genes, with *COQ4* having the highest mutation rate owing to the Chinese-specific founder mutation (4/66, 6%). Twelve patients (12/66, 18%) had mutations in non-MD-related genes: *ATP1A3* (*n* = 3, two were siblings), *ALDH5A1*, *ARX*, *FA2H*, *KCNT1*, *LDHD*, *NEFL*, *NKX2-2*, *TBCK*, and *WAC*.

**Conclusions:**

We confirmed that the *COQ4*:c.370G>A, p.(Gly124Ser) variant, was a founder mutation among the Southern Chinese population. Screening for this mutation should therefore be considered while diagnosing Chinese patients suspected to have MDs. Furthermore, WES has proven to be useful in detecting variants in patients suspected to have MDs because it helps to obtain an unbiased and precise genetic diagnosis for these diseases, which are genetically heterogeneous.

## Background

Mitochondrial diseases (MDs) are a common group of inborn errors of metabolism (IEMs) that are caused by defects in oxidative phosphorylation and have an estimated birth prevalence of 1:5000 [1] and premature mortality rate of 14% [2]. Inherited and de novo pathogenic variants have been reported in 37 mitochondrial genes (i.e. in mtDNA) and 295 nuclear genes (i.e. in nDNA) [3]. The clinical manifestations of MDs are highly variable and indicate organ-specific or multi-systemic involvement [4]. MDs are difficult to diagnose because of their clinical and genetic heterogeneity. Involvement of novel genes, mitochondrial mimicry, and tissue specificity of mtDNA heteroplasmy further complicate the diagnosis [5].

A consensus statement on the diagnosis and management of MDs was published in 2015. As per this statement, patients suspected to have MDs should undergo clinical evaluation along with biochemical testing of the blood, urine, and cerebrospinal fluid. For investigating mtDNA mutations, deletion, duplication, and depletion, next-generation sequencing (NGS)-based methods were suggested. Furthermore, it stated that muscle or liver samples should also be assessed in cases where negative results were obtained with blood samples. The statement provides limited information on the genetic testing of nDNA. Gene panel-based NGS was listed as the preferred method for nDNA testing. If the NGS results are negative, whole-exome sequencing (WES) should be considered. Tissue biopsies and subsequent electron transport chain (ETC) enzymology should be performed only when DNA testing cannot provide a diagnosis [6].

Despite the recommendations provided in the consensus statement, multiple subsequent studies used WES or NGS for diagnosing MDs. The cohort of Wortmann et al. [7] included 109 patients up to 27 years of age who had no pathogenic variants detected by mtDNA investigations. Data were first filtered using a virtual MD gene panel comprising 238 genes. If the results were negative, WES was performed. The overall diagnostic yield was 38% (42/109), with an equal number of diagnoses being obtained for cases involving MD-related and non-MD-associated genes (19%, 21/109). Pronicka et al. [8] used WES for a cohort of 113 paediatric patients who had MDs and in whom pathogenic variants were not found. The diagnostic rate was 59% (67/113), with 17% of the pathogenic nDNA variants (19/113) being identified in non-MD-associated genes. Kohda et al. [9] and Theunissen et al. [10] both used NGS-based mtDNA sequencing followed by WES. In the cohort of Kohda et al. [9] of 142 patients with childhood-onset mitochondrial respiratory chain complex deficiencies, the WES diagnostic yield was 25% (35/142). WES helped diagnose 49% (57/117) of the patients in the study by Theunissen et al. [10], in which 86 and 31 patients were suspected to have MDs and neuromuscular diseases, respectively.

To summarize, six studies during 2014–2019 suggested using WES and/or NGS-based mtDNA sequencing over panel-based diagnostic methods for patients suspected to have MDs, on the basis of the high diagnostic yields (35–66%) of the former approach [5, 7–11]. The diagnostic yield will be higher if there is a high proportion of familial and consanguineous cases [11], if the patients have decreased activities of multiple respiratory chain complexes [8], in neonatal-onset cases [7], and in cases with severe presentation [12]. This was further supported by the findings of Pronicka et al. [8] wherein the percentage of positive WES results increased with increase in the mitochondrial disease criteria (MDC) score of patients.

In the case of MD genes, there was very little overlap across five studies during 2014–2018. Only 11 known MD nuclear genes, i.e. *SCO2*, *MTFMT*, *TAZ*, *NDUFS7*, *RRMB2B*, *MTO1*, *EARS2*, *RARS2*, *C12orf65*, *PC*, and *FBXL4*, were identified in three or more studies [7–11]. WES-positive findings were obtained in a high percentage of cases involving non-MD genes. Up to 19% of the cohort in a study by Wortmann et al. [7] was found to have mutations in non-MD genes. These findings show that gene panels will be inadequate for identifying variants in novel genes or non-MD genes.

Founder mutations have been identified in certain ethnic populations. Recurrent rare variants found in *FBLX4*, *ACAD9*, and *CLPB* in Polish patients with MDs suggested the ethnic specificity of these variants [8].

Therefore, in the current study, using WES, we aimed to identify the disease-causing mutations in patients who were of a Chinese descent and had paediatric-onset MDs. The findings provide improved understanding of variants and their prevalence in the Chinese population, which may have been underrepresented in previous studies.

## Results

### Clinical presentation

We recruited 66 patients, including 42 male and 24 female individuals (male to female ratio, 1.75:1). The age of onset ranged from the neonatal period to 9 years, with the median age of onset being 1.75 years of age. Nineteen patients presented at the neonatal stage. The patients recruited were from 63 non-consanguineous Chinese families, with a positive family history being found in five cases (siblings = 3 cases, siblings and mother = 1 case, paternal cousin = 1 case). There were 18 and 48 cases of syndromic and non-syndromic MD, respectively. The most frequently identified clinical feature was global developmental delay (GDD; 50%; 33/66), followed by epilepsy/seizure (44%; 29/66) and dystonia (40%, 26/66). Myopathy was noted in 27% of the cohort (18/66), and 23% and 18% of the patients had visual issues (15/66) and oromotor dysfunction (12/66), respectively. Cardiopulmonary, auditory, renal, and hepatic issues were less common. Neuroimaging was performed for 65 patients; of these patients, 50 had abnormal neuroimaging features. Among all the anomalies, features including signal changes in the basal ganglia (18%; 12/66), cerebral or cerebellar atrophy (15%; 10/66), and optic atrophy (2%; 1/66) were noteworthy. Furthermore, 36% of the cohort (24/66) had lactate acidosis. For clinical evaluation, Mitochondrial Disease Criteria (MDC) were used to guide and standardize the diagnosis for paediatric-onset MD. This score is based on various clinical criteria including muscular presentations, central nervous system (CNS) abnormalities, multisystemic involvement, metabolic and neuroimaging studies, and histological morphology. Each case can be classified into unlikely, possible, probable, or definite MD subgroups [13]. On the basis of the pre-biopsy MDC score, 14% (9/66) of the patients were classified into the possible, 38% (25/66) into the probable, and 48% (32/66) into the definite MD subgroups. Mitochondrial enzyme analysis was performed for 45 patients, with muscle (*n* = 40), skin (*n* = 39), and liver (*n* = 1) being used as tissue sources. The most common deficiency was a complex IV deficiency (13%; 6/45; five from muscle and one from muscle and skin), followed by complex II + III (11%; 5/45; all from skin), complex I + IV (7%; 3/45; all from muscle), and complex I (4%; 2/45; one from muscle and one from skin) deficiencies. Muscle biopsy was performed for 40 patients (61%); of these, seven showed an increase in MDC score after the biopsy sample analysis, but only one patient was re-classified from the probable to the definite MD subgroup.

### Molecular findings

WES was performed for 55 patients as singletons, four as duos (three pairs of affected siblings and an affected mother-child pair), and seven as trios. Segregation analysis was performed after WES for 16 singletons and two siblings.

**Fig. 1:**
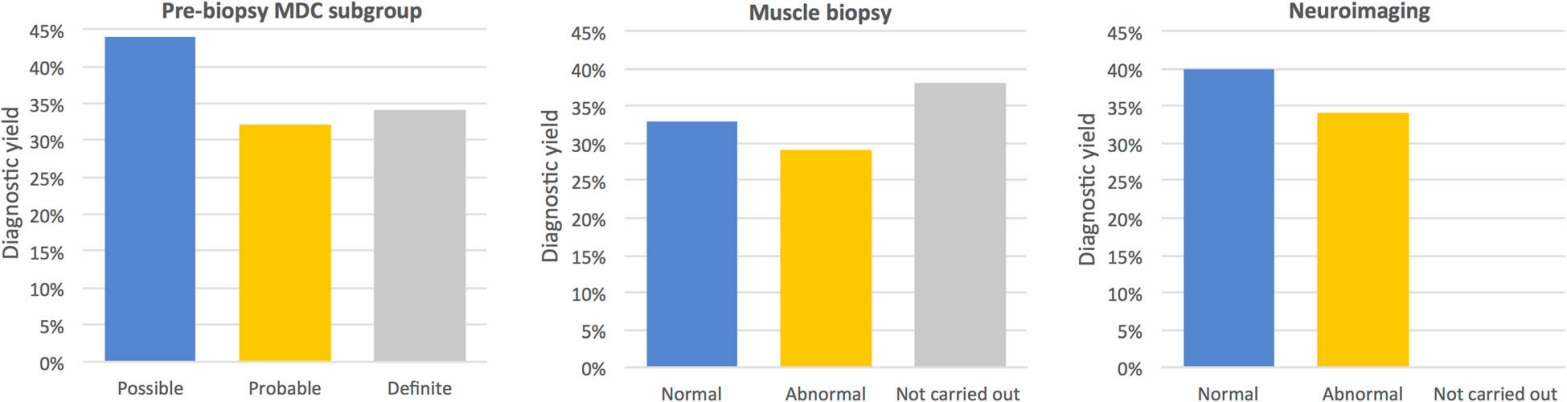
Diagnostic yield according to the MDC subgroup, muscle biopsy findings, and neuroimaging findings

Disease-causing variants were detected in 10 patients by using singleton WES analysis. Segregation analysis helped classify the variants in 12 cases as pathogenic or likely pathogenic. The reanalysis performed in 2019 helped diagnose one more case in our cohort, leading to an overall diagnostic yield of 35% (23/66). Muscle biopsy was not required in any of the cases to confirm the pathogenicity of the variant. In one case, the pathogenic variant in the *OPA1* gene did not fully explain the phenotype because the patient did not have an optic atrophy by that time and he had more severe manifestations beyond optic problems, with generalized dystonia and bilateral basal ganglion lesions being noted in neuroimaging studies (patient 11). The WES findings for two patients (patient 10 and patient 6) showed disease-causing variants in *OPA1* and *SURF1*, respectively, and these findings were correlated with their corresponding syndromic presentations of autosomal dominant optic atrophy (ADOA) and Leigh syndrome. The highest diagnostic yield was obtained in the possible MD subgroup (44%; 4/9), followed by the definite (34%; 11/32) and probable MD (32%; 8/25) subgroups. Patients with either normal muscle biopsy or normal neuroimaging results had a higher diagnostic yield (33% [11/33] and 40% [6/15], respectively) than patients with abnormal findings (Fig. 1). Pathogenic variants were identified in 37% (7/19) of the neonatal cases.

In our first-tier analysis using MitoCarta, 11 patients were found to have mutations in genes included in the panel. In our second-tier open-exome analysis, 12 more patients were diagnosed. Eleven patients (17%) were found to carry disease-causing mutations in six MD-related genes, i.e. *COQ4*, *COQ7*, *NDUFA9*, *OPA1*, *SURF1*, and *TAZ*. Twelve patients (18%) had mutations in 10 non-MD-associated genes, i.e. *ATP1A3*, *ALDH5A1*, *ARX*, *FA2H*, *KCNT1*, *LDHD*, *NEFL*, *NKX2-2*, *TBCK*, and *WAC*. From all the genes identified (MD and non-MD), five showed autosomal dominant inheritance, nine showed autosomal recessive inheritance, and two showed X-linked inheritance. The recurrent disease-causing genes were *COQ4* (*n* = 4), *OPA1* (*n* = 2), and *ATP1A3* (*n* = 3, two from siblings). The findings are summarized in Tables 1 and 2.

**Table 1:** Clinical presentation and disease-causing variants identified in patients with MDs

Patient no./sex	Family history	Age of onset/age of last follow-up	Initial presentation	Muscular	CNS	Heart	Vision	Hearing	Neuro-imaging	MDC score (pre-biopsy)	Gene	Variant	Disease association
1/M	−	Day 1/8 mo^a^	Non-syndromic	+	+	+	−	+	+	8, definite	*COQ4*	c.370G>A, p.(Gly124Ser), and c.402+1G>C	Coenzyme Q10 deficiency, primary, 7
2/F	−	Infancy/4 yo	Non-syndromic	−	+	−	−	−	+	6, probable	*COQ4*	c.371G>T, p.(Gly124Val), and c.370G>A, p.(Gly124Ser)	Coenzyme Q10 deficiency, primary, 7
3/M	−	Infancy/3 yo	Non-syndromic	−	+	−	−	−	+	6, probable	*COQ4*	c.402+1G>A and c.550T>C, p.(Trp184Arg)	Coenzyme Q10 deficiency, primary, 7
4/F	−	Birth/3 yo^a^	Non-syndromic	−	+	−	−	+	+	8, definite	*COQ4*	c.370G>A, p.(Gly124Ser) homozygous	Coenzyme Q10 deficiency, primary, 7
5/M	−	Birth/1 yo^a^	Non-syndromic	−	+	−	−	+	+	8, definite	*COQ7*	c.319C>T, p.(Arg107Trp), and c.599_600delinsTA ATGCATC, p.(Lys200Ilefs*56)	Coenzyme Q10 deficiency, primary, 8
6/F	−	Infancy/24 yo	Syndromic	−	+	−	−	−	+	8, definite	*SURF1*	c.792_793del, p.(Arg264Serfs*27), and c.529del, p.(Val177*)	Leigh syndrome, due to COX IV deficiency; Charcot–Marie–Tooth disease, type 4 K
7/M	−	8 yo/43 yo	Non-syndromic	−	+	−	−	−	+	5, probable	*NDUFA9*	c.1078C>T, p.(Arg360Cys) homozygous	Mitochondrial complex I deficiency, nuclear type 26
8/M	+ (sibling of patient 9)	Neonatal/18 yo	Syndromic	+	−	+	−	−	+	7, probable	*TAZ*	c.718G>C, p.(Gly240Arg) hemizygous	Barth syndrome
9/M	+ (sibling of patient 8)	5 months/9 yo	Syndromic	−	+	+	−	−	−	4, possible	*TAZ*	c.718G>C, p.(Gly240Arg) hemizygous	Barth syndrome
10/M	−	4 yo/8 yo	Syndromic	−	−	−	−	−	+	3, possible	*OPA1*	c.1218delC, p.(Glu408Lysfs*7)	Optic atrophy 1; optic atrophy plus syndrome
11/M	−	Infancy/7 yo	Non-syndromic	−	+	−	−	−	+	6, probable	*OPA1*	c.2345A>G, p.(His782Arg)	Optic atrophy 1; optic atrophy plus syndrome

*M* male, *F* female, *mo* month(s), *yo* year(s), *CNS* central nervous system

^a^Age at death

**Table 2:** Clinical presentation and disease-causing variants identified in non-MD patients

Patient no./sex	Family history	Age of onset/age of last follow-up	Initial presentation	Muscular	CNS	Heart	Vision	Hearing	Neuro-imaging	MDC score (pre-biopsy)	Gene	Variant	Disease association
12/M	+ (mother, sibling of patient 13)	8 mo/24 yo	Non-syndromic	+	+	−	−	−	+	8, definite	*ATP1A3*	c.2452G>A, p.(Glu818Lys)	Alternating hemiplegia of childhood 2; CAPOS syndrome; Dystonia-12
13/F	+ (mother, sibling of patient 12)	7 mo/26 yo	Non-syndromic	+	+	−	−	−	−	8, definite	*ATP1A3*	c.2452G>A, p.(Glu818Lys)	Alternating hemiplegia of childhood 2; CAPOS syndrome; Dystonia-12
14/M	−	Birth/14 yo	Non-syndromic	+	+	−	−	−	−	8, definite	*ARX*	c.989G>A, p.(Arg330His) hemizygous	Epileptic encephalopathy, early infantile, 1; hydranencephaly with abnormal genitalia; Lissencephaly, X-linked 2; Mental retardation, X-linked 29 and others; Partington syndrome; Proud syndrome
15/F	+ (paternal cousin)	Birth/14 yo	Non-syndromic	+	+	−	−	−	−	7, probable	*ATP1A3*	c.2755_2757del GTC, p.(Val919del)	Alternating hemiplegia of childhood 2; CAPOS syndrome; Dystonia-12
16/M	−	Infancy/4 yo	Non-syndromic	+	+	−	−	−	+	8, definite	*WAC*	c.1171dupA, p.(Thr391Asnfs*15)	Desanto-Shinawi syndrome
17/M	−	3.5 yo/9 yo	Non-syndromic	−	+	−	−	−	+	8, definite	*ALDH5A1*	c.515G>A, p.(Arg172His) homozygous	Succinic semialdehyde dehydrogenase deficiency
18/F	−	9 yo/29 yo	Non-syndromic	−	+	−	−	−	+	5, probable	*FA2H*	c.460C>T, p.(Arg154Cys) homozygous	Spastic paraplegia 35, autosomal recessive
19/M	−	Infancy/26 yo	Non-syndromic	+	+	−	−	−	−	5, probable	*NEFL*	c.293A>G, p.(Asn98Ser)	Charcot–Marie–Tooth disease, dominant intermediate G; type 1F; type 1E
20/M	−	Neonatal/8 yo^a^	Non-syndromic	+	+	−	−	−	+	8,definite	*KCNT1*	c.1420C>T, p.(Arg474Cys)	Epilepsy, nocturnal frontal lobe, 5; Epileptic encephalopathy, early infantile, 14
21/F	−	Infancy/30 yo	Non-syndromic	+	+	−	−	−	+	8, definite	*NKX2-2*	c.32C>T, p.(Ser11Leu) homozygous	Diabetes—neonatal onset
22/M	−	Infancy/10 yo^a^	Non-syndromic	+	+	−	−	−	+	4, possible	*TBCK*	c.976dupT, p.(Tyr326Leufs*10) and c.478G>T, p.(Glu160*)	Hypotonia, infantile, with psychomotor retardation and characteristic facies 3
23/F	−	Infancy/13 yo	Non-syndromic	−	+	−	−	−	−	4, possible	*LDHD*	c.469+1dup and c.752C>T, p.(Thr251Met)	D-lactic aciduria

*M* male, *F* female, *mo* month(s), *yo* year(s), *CNS* central nervous system, *CAPOS* cerebellar ataxia, areflexia, pes cavus, optic atrophy, and sensorineural hearing loss syndrome

^a^Age at death

The gene showing the highest number of recurrent mutations was *COQ4* (*n* = 4), with the c.370G>A, p.(Gly124Ser) variant, being found in three patients. Patients with the *COQ4* mutation can present with symptoms at either neonatal or infantile stages. Clinical presentations including GDD, seizures, generalized dystonia, bilateral cortical visual impairment, oromotor dysfunction, hearing problems, and lactate acidosis were noted in our study. Basal ganglion changes and cerebral atrophy were observed on analysing the magnetic resonance imaging (MRI) findings of patients with *COQ4* mutations.

## Discussion

In this study, WES was performed for 66 Chinese patients with paediatric-onset MDs. Despite the lack of a local diagnostic facility for a respiratory chain enzyme analysis, an overall diagnostic yield of 35% (23/66) was achieved, which was comparable to that of similar studies in recent years [7–11]. Among the cases identified by WES, 48% (11/23) had mutations in MD genes, while the remaining (12/23) had mutations in non-MD genes.

### *COQ4* mutation was the most important cause of MDs caused by nDNA mutations in the Southern Chinese patients

Eleven patients (17%) were found to have mutations in MD-related genes, with *COQ4* having the highest mutation rate (4/66, 6%). The *COQ4* missense variants were classified as VUSs at the initial stage of this study. The pathogenicity was supported by the segregation study and functional analysis of the skin fibroblasts, which showed complex II + III deficiency and low CoQ concentration. *COQ4* is associated with primary coenzyme Q10 deficiency type 7 [14]. Subsequent to the first Chinese study published in 2019 by Lu et al. [15], which described a pair of siblings with Leigh syndrome caused by homozygous *COQ4* c.370G>A, p.(Gly124Ser), our team published the largest *COQ4* cases series with 11 Chinese patients from nine families (including patients 1 to 4 in this study) in collaboration with a Taiwanese group [16]. Our collaborative study proved that CoQ10 deficiency could occur in neonates, infants, or children with variable phenotypes, which altered the original notion that CoQ10 deficiency shows only neonatal onset [14, 17]. Furthermore, we found that 10 of the 11 patients carried the c.370G>A, p.(Gly124Ser) mutation. Using a single nucleotide polymorphism (SNP) array, we have previously shown that this mutation is a Chinese-specific founder mutation. In the same year of 2019, Ling et al. [18] also described three unrelated Chinese patients with this founder mutation. Currently, 16 Chinese *COQ4* cases have been reported, with 15 patients in 12 non-consanguineous Chinese families carrying the c.370G>A Chinese founder mutation.

Performing multiple studies with patients from the same ethnic background can aid in effectively identifying recurrent founder mutations. In a study by Piekutowska-Abramczuk et al. [19], the c.845_846delCT variant was found in 77.6% of the *SURF1* alleles in the cohort of Polish patients; however, it was found in a much lower percentage (9%) in the non-Polish population. In 2013, Pronicka et al. [20] studied a cohort with a similar ethnic background; a homozygous c.1541G>A variant in *SCO2* (a known MD gene) was identified in an extremely high proportion of the cohort (35/36). In 2016, the detection of recurrent rare pathogenic variants of *FBXL4*, *ACAD9*, and *CLPB* further extended the scope of the suspected Polish-specific MD mutations. The ethnic specificity (Polish) observed in the MD-causing genes [8] parallels the situation of this study in which the *COQ4* variant is strongly enriched in cohorts of Chinese patients. A recent study on IEMs have also identified other founder mutations, e.g. *GCDH* and *SLC25A20*, in the Southern Chinese population [21]. It is postulated that, when more cases are identified in the Chinese population, the frequency of the c.370G>A, p.(Gly124Ser) variant, or other MD-causing mutations will also subsequently increase. Therefore, we suggest that more studies should be performed in Chinese populations to identify any ethnic-specific variants in MD genes.

### Benefits of using WES for first-tier genetic testing

According to the 2015 consensus statement [6], patients should undergo WES only if positive results are not obtained with targeted gene sequencing, gene panel analysis, and mtDNA sequencing. However, in our study, as well as in several previous studies [7–11], WES was chosen as the diagnostic method instead of gene panel-based NGS because of the sensitivity of WES. On comparing genes identified in our first-tier results with genes on MitoCarta, we found that 56% (9/16; one MD and eight non-MD genes) of the genes identified in our study were not included in the MitoCarta gene panel. Furthermore, 44% (7/16) of the genes would have been missed if the mitochondrial disorders panel of Genomics England PanelApp (gene panel curated by UK Genetic Testing Network–Association for Clinical Genome Science) [22] would have been used (seven non-MD genes). If a panel-based NGS method had been adopted instead of WES, 52% (12/23) of the patients found to be positive by WES would not have been identified in our study. This statistic is backed by four studies that were conducted after 2015 and used WES [7–10], wherein 24–49% of the genes identified would have been missed if gene panel-based NGS based on MitoCarta had been used. The false-negative rates of MitoCarta panel-based NGS range from 16 to 45%.

We compared our study with five other studies that were performed during 2014–2018 and used WES for MD diagnosis [7–11]. A total of 92 MD genes were identified in those five studies; however, only 31 (34%) of these genes were reported more than once. Among those 31 genes, only five were also reported in our study indicating that there was minimal overlap (Table 3). More importantly, all studies identified the disease-causing mutations in non-MD genes in patients with suspected MDs. In our study, 12 patients (18%) had non-MD gene mutations. Non-MD gene involvement has also been significant in previous studies [7–11], ranging from 2 to 19%, but only *MECP2* was reported in more than one of the five studies published during 2014–2018 (Table 4). Non-MD genes would not have been detected if only gene panel-based NGS, e.g. MitoCarta, had been used. We therefore recommend WES as a first-tier genetic test for patients with MDs because of the high genetic heterogeneity noted in such cases.

**Table 3:** MD genes that appeared more than once in six studies, i.e. the current study and five previous studies [7–11]

Gene	Disease-causing genes found in				
	Current study	Number of previous studies			
		Four	Three	Two	One
***TAZ***^***#***^	v		v		
***SCO2***		v			
***MTFMT***		v			
***NDUFS7***			v		
***RRM2B***^***#***^		v			
***MTO1***			v		
***EARS2***			v		
***RARS2***			v		
***C12orf65***			v		
***PC***				v	
***FBXL4***			v		
***COQ4***	v				v
***COQ7***	v				v
***OPA1***	v				v
***SURF1***	v				v
***NDUFV1***				v	
***TMEM126B***				v	
***ACAD9***				v	
***SLC25A4***				v	
***TRMU***				v	
***KARS***				v	
***VARS2***				v	
***AARS2***				v	
***GFM1***				v	
***SERAC1***				v	
***MFN2***				v	
***COX10***				v	
***SLC19A3***^***#***^				v	

^#^Absent in MitoCarta 2.0

**Table 4:** Non-MD genes detected by WES and the diagnostic yield in non-MD genes in five studies (including this study)

Categorization	Current study	Theunissen et al. [10]	Kohda et al. [9]	Pronicka et al. [8]	Wortmann et al. [7]
**Neuronal diseases**	*ATP1A3 ALDH5A1 ARX FA2H KCNT1 NEFL NKX2-2 TBCK WAC*	*IER3IP1 IARS CHRNE SLC16A2*	***MECP2***	*ADAR CACNA1A CLN3 DMD DYSF GBE1 GFAP HSD17B4* ***MECP2*** *MYBPC1 PGAP2*	*ARID1B SCN1A ASPM CTNNB KDM6A SMARCA4 SETBP1 ACTA1 NGLY1 ALDH4A1 RAPSN COL4A1 TBR1*
**Eye diseases**		*HPS1*			
**Metabolic diseases**	*LDHD*				
**Haematological diseases**		*BICD2*		*CPS1 PRF1 SBDS*	
**Cardiological diseases**			*TNNI3*		
**Nephrological disease**				*PIGN*	*SLC3A1*
**Endocrine diseases**	*NKX2-2*				
**Not found in GeneAnalytics**				*PEXS*	*CTNNB SEPN1*
**Diagnostic yield in non-MD genes**	18% (12/66)	6% (5/86)	2% (3/142)	17% (19/113)	19% (21/109)

Categorization was based on GeneAnalytics [23]. *MECP2* is the only non-MD-associated gene that has appeared in more than one study

Although analysis of the respiratory chain enzyme activity may provide an effective approach for screening out potential patients with MDs, this test is currently unavailable in Hong Kong. Furthermore, the protocol is not standardized, which may lead to discrepancies because of variation in the assay conditions and control values used by different laboratories [24]. The use of WES for first-tier analysis can help avoid unnecessary invasive biopsies. In our study, 20% of such procedures could have been avoided in one MD and eight non-MD cases because molecular diagnosis was directly established through WES. Furthermore, secondary complex IV deficiency was detected in two non-MD patients with *ARX* and *LDHD* mutations. These findings indicate that WES is a more comprehensive diagnostic approach than the use of enzymology alone to diagnose MDs. On the other hand, the traditional way of initially investigating a patient suspected of a MD biochemically followed by WES might reveal enzymatic OXPHOS defects in non-MD genes previously linked to well-defined syndromes but not brought into association with mitochondrial failure before. Understanding such pathophysiology with functional studies would hopefully close this knowledge gap. The lack of clear phenotype–genotype relationships and the fact that ongoing studies constantly identify novel mutations make it inefficient to determine what additional gene panel(s) should be used in conjunction with an MD gene panel. This is because non-MD-associated genes could be involved. The use of WES can help fill in existing knowledge gaps and prevent delay in achieving an accurate genetic diagnosis.

With the improvements achieved in bioinformatics analysis, mitochondrial DNA variants can be identified using the off-target read from WES with high recall rate and precision [5]. Overall, the findings indicate that WES should be used for first-tier genetic testing in our local setting. We also propose that WES results should be incorporated in the diagnostic criteria of MDs.

## Limitations of our study and future directions

There are certain shortfalls of using WES. Pathogenic variants located in the non-coding region could not be detected. Single/multiple deletions, or depletion of mtDNA, and low heteroplasmy variants could have been missed [5]. With technological improvements, it is expected that whole-genome sequencing (WGS) will also be used in the future, when reductions in cost and increased accuracy of variant interpretation are achieved [3].

## Conclusions

To our knowledge, this is the largest WES study on Chinese paediatric-onset MDs. Primary coenzyme Q10 deficiency type 7 was found to be the most common MD caused by nDNA mutations in our cohort, owing to the Chinese founder mutation, c.370G>A, p.(Gly124Ser). Clinicians should actively consider the possibility of *COQ4* mutation and provide biochemical and genetic workups for Chinese patients suspected to have MDs. Owing to the highly variable phenotypes and genes involved in paediatric-onset MDs, we recommend the use of WES as a first-tier test to maximize the diagnostic yield. The molecular diagnoses obtained using WES will help to guide diagnosis and management and should be incorporated into future diagnostic criteria of MDs; this is especially relevant in geographical regions where enzyme testing is not available.

## Methods

### Patient recruitment

A total of 66 patients under clinical management at the Queen Mary Hospital and the Duchess of Kent Children’s Hospital, which are affiliated with the University of Hong Kong (HKU), were recruited during 2011–2018. The inclusion criteria were as follows: (1) suspicion of MDs by the study authors (F.C.W. and C.H.Y.B.), along with a minimum pre-biopsy MDC score of 3 (MD subgroups: possible, score of 3–4; probable, score of 5–7; or definite, score of 8–12) (13); (2) absence of large-scale mtDNA deletions and recurrent point mutations in the mitochondrial genome; and (3) normal chromosome microarray results.

The cohort is representative of the heterogeneous patient population noted during genetic and neurometabolic consultations provided by the authors as part of the Hong Kong health care system, which ranged from neonatal intensive care unit (NICU) cases to out-patient cases.

### Clinical evaluation

Clinical evaluation was performed as per the recommendations of our paediatric neurologist (F.C.W.) and clinical geneticist (C.H.Y.B.). Most of the clinical, metabolic, histological, electrophysiological, and radiological analyses were performed locally. The only exception was the analysis of oxidative phosphorylation enzyme activities (OXPHOS) in freshly obtained muscle and fibroblast samples from our cohort, which was performed at the Radboud Center for Mitochondrial Medicine, Nijmegen [25], as such technology is unavailable in Hong Kong. During the same period of 2011–2018, three other patients were diagnosed with MDs by using a molecular diagnostic approach in the routine clinical setting and were therefore excluded from the study. These patients included two with the *MT-ND5* m.13513G>A mutation and one with the *MT-ND5* m.13052G>A mutation, presenting as mitochondrial encephalopathy, lactic acidosis, and stroke-like episodes (MELAS; *n* = 2) and non-syndromic MD, respectively.

### Whole-exome sequencing

Genomic DNA was extracted from peripheral blood samples by using the Qiagen Blood Mini Kit (Qiagen). For the first 41 patients, WES was performed at Genome Diagnostics, Radboud University, Nijmegen, using previously described methodology [7]. For the subsequent 25 cases, WES was performed at the Department of Paediatric and Adolescent Medicine of HKU [26]. The exome library was prepared using the TruSeq Rapid Exome Library Prep Kit (Illumina) and sequenced using the Illumina NextSeq500 system. The in-house bioinformatics pipeline was used for data analysis. Raw reads were aligned to the reference human genome [hg19] with the Burrows–Wheeler Aligner (BWA) 0.7.10 [27] and processed following the Genome Analysis Toolkit (GATK) best practices [28]. Rare variants were analysed for pathogenicity according to the American College of Medical Genetics and Genomics (ACMG) guidelines [29]. Data analysis consisted of an initial filtering step involving a virtual MD gene panel based on MitoCarta 2.0; this panel consisted of 1158 genes which encode proteins that have been strongly correlated with mitochondrial localization [30]. If the results were negative, the entire exome was analysed. This approach would include analysis on secondary MD-associated genes and non-MD-associated genes, and the latter have also been reported to be involved in MD in previous studies [7–10]. For variants of uncertain significance (VUSs), the phenotype was reassessed in the same way in order to curate possible novel disease-causing variants. The data for the initially negative WES results were reanalysed in July 2019 after considering the findings of other recent studies on MDs. The subsequent variant confirmation and segregation analysis were performed at our department in HKU.

## Data Availability

The datasets used and/or analysed during the current study are available from the corresponding author on reasonable request.
